# A Small-Scale Field Trial of Pyriproxyfen-Impregnated Bed Nets against Pyrethroid-Resistant *Anopheles gambiae* s.s. in Western Kenya

**DOI:** 10.1371/journal.pone.0111195

**Published:** 2014-10-21

**Authors:** Hitoshi Kawada, Gabriel O. Dida, Kazunori Ohashi, Emiko Kawashima, George Sonye, Sammy M. Njenga, Charles Mwandawiro, Noboru Minakawa

**Affiliations:** 1 Department of Vector Ecology and Environment, Institute of Tropical Medicine, Nagasaki University, Nagasaki, Japan; 2 School of Public Health, Maseno University, Kisumu, Kenya; 3 Health and Crop Sciences Research Laboratory, Sumitomo Chemical Co. Ltd., Hyogo, Japan; 4 Springs of Hope, Mbita, Kenya; 5 Eastern and Southern Africa Centre of International Parasite Control, Nairobi, Kenya; 6 Kenya Medical Research Institute, Nairobi, Kenya; 7 The Global Center of Excellence Program, Nagasaki University, Nagasaki, Japan; Ehime University, Japan

## Abstract

Pyrethroid resistance is becoming a major problem for vector control programs, because at present, there are few suitable chemical substitutes for pyrethroids, as when used on bed nets the insecticide must have low mammalian toxicity as well as high activity to mosquitoes. Pyriproxyfen (PPF) is one of the most active chemicals among the juvenile hormone mimic (JHM) group. Sterilizing mosquitoes by using PPF could be a potential control measure for pyrethroid-resistant malaria vectors. We investigated the sterilizing effects of two types of PPF-impregnated bed nets – a 1% PPF-impregnated net and a 1% PPF +2% permethrin-impregnated net (Olyset Duo) – to pyrethroid-resistant wild population of *Anopheles gambiae* s.s. in western Kenya. High mortality of blood-fed mosquitos was observed 3 days post-collection, in the houses where PPF-impregnated nets were used, indicating the effect of PPF on the longevity of mosquitos that came in contact with the net. Reduction in the number of ovipositing females, number of eggs, and number of progeny per female were also observed in the houses in which both Olyset Duo and PPF-impregnated nets were used. This is the first field study showing the high sterilizing efficacy of PPF against wild pyrethroid-resistant *An. gambiae* s.s. population. In addition, we recognized the necessity of combined use of permethrin with PPF, in order to reduce the risk of mosquito bites and provide a level of personal protection. Further studies on wild pyrethroid-resistant mosquito populations such as *An. arabiensis* and *An. funestus* s.s. would provide more information on the practical use of the PPF-impregnated bed nets.

## Introduction

After more than 30 years since the introduction of permethrin, synthetic pyrethroids have emerged as the newest class of insecticides in the vector control program [1]. On a global level, pyrethroids constitute approximately 81% of the spray utility, among which 68% is used for residual spraying and 24% for space spraying, and 100% of WHO-recommended insecticides for the treatment of long lasting mosquito nets (LNs) [2]. The resistance of vector mosquitoes is one of the main obstacles in effective vector control, because currently, there are no suitable substitutes for pyrethroids [1]. More than 90% of the current annual malaria incidence occurs in Africa. *Anopheles gambiae* Giles (*An. gambiae* s.s.), *Anopheles arabiensis* Patton and *Anopheles funestus* Giles (*An. funestus* s.s.) are the primary malaria vectors in sub-Saharan Africa. Recently, a causal relationship between the high coverage of LNs due to mass campaigns and the increase in the *kdr* frequency in *An. gambiae* s.s. have been reported [3], [4], [5]. In contrast, such *kdr* mutations do not seem to be common in *An. arabiensis* [6]–[8], but the metabolic resistance seems to be most common in this species [8], [9]. Fortunately, *kdr* mutations have not been reported in African malaria vectors except in these two species. *Anopheles funestus* s.s. is the major malaria vector in southern Africa. Re-emergence or increase of this species as well as the development of metabolic pyrethroid resistance in eastern and southern Africa have been reported in this species [9]–[15].

Juvenile hormone mimics (JHMs) developed from natural origins are one of the commercially successful chemicals in the insect growth regulators (IGRs) category, with a unique mode of action that is insect-specific, stage-specific, slow acting, and non-neurotoxic [16]. Methoprene [17] and pyriproxyfen [18] are the most successful JHMs. Such chemicals have been commonly used as larvicides, since most of the effects of JHMs are on the last instar larvae, which become deformed or die at the pupal stage [18]. Pyriproxyfen was reported to cause vacuolation, inhibited development of imaginal buds of *Aedes aegypti* (L) larvae, and histolysis such as disrupted mitochondria, abundant vacuoles and poorly structured cytoplasmic organelles [19]. Adult *An. balabacensis* Baisas, which survived 48 h of immersion in 0.005 ppb (one-eighth of LC_50_) of pyriproxyfen during the last larval instar, was found to show considerable reduction in sperm and egg production, as well as in blood feeding and mating activity [20].

JHMs also acted as a “sterilant” in adult insects. Methoprene was reported to affect ovarian development and adult longevity in *Ae. aegypti* [21], [22]. The number of eggs per female and hatchability decreased with the application of pyriproxyfen to female *Ae. aegypti* [23], [24], housefly (*Musca domestica* L.), and German cockroach (*Blattella germanica* L.) [25]. Aiku et al. [26] first demonstrated the use of pyriproxyfen-impregnated bed net as a sterilizing device for Anopheline mosquitoes. Ohashi et al. reported that the adult females of insecticide-susceptible *An. gambiae* strain were completely sterilized after exposure to 0.01% pyriproxyfen-treated nets (3.5 mg active ingredient [AI]/m^2^) before and after consuming blood. It was also seen that the adult longevity decreased by exposure to pyriproxyfen in a dose-dependent manner [27]. Similarly, Harris et al. reported that the laboratory colony of *An. arabiensis*, which blood-fed one day prior to pyriproxyfen exposure (3 mg [AI]/m^2^), produced no viable offspring, while other treatments (blood-fed 3 days before exposure, 1 day after exposure, and 3 days after exposure) had no significant effect [28].

The above findings suggested that sterilizing technique using pyriproxyfen could be one of the most effective measures for controlling pyrethroid-resistant malaria vectors. However, the field conditions in which people use bed nets and the wild population of malaria vectors come into contact with the bed nets voluntarily, have not been thoroughly studied. Here, we assessed the sterilizing effects of two types of pyriproxyfen-impregnated bed nets −1% pyriproxyfen-impregnated net and 1% pyriproxyfen +2% permethrin-impregnated net (Olyset Duo) – on a pyrethroid-resistant wild population of *An. gambiae* s.s. in western Kenya.

## Materials and Methods

### Bed nets

Three kinds of bed nets were provided by Sumitomo Chemical Co. Ltd. (Tokyo, Japan). The first one was impregnated with 1% pyriproxyfen (PPF), the second with 1% pyriproxyfen and 2% permethrin (Olyset Duo), and the third with 2% permethrin (Olyset Net). All bed nets were of the same size (130 cm [W]×180 cm [L]×150 cm [H]) and were made of polyethylene netting material (mesh 20 holes/cm^2^), with active ingredients incorporated into the polymer before monofilament yarn extrusion.

### Study location

The study was conducted in Ragwe village, in the Suba south sub-county in Homabay county of Nyanza Province in Western Kenya. The Suba south sub-county has been identified as a high malaria transmission area in Kenya, with more than 50% of the population exposed to ≥40% *Plasmodium falciparum* parasite rate corrected to a standard age range of 2 years old to <10 years old [29]. The main malaria vectors in the area are *An. gambiae* s.s., *An. arabiensis*, and *An. funestus* s.s. *Anopheles rivulorum* Leeson, which belongs to the Funestus Group is a minor vector in that area [12]. These four species were recently reported to have developed multimodal pyrethroid resistance [9], [12]. *Anopheles gambiae* s.s. mainly dominated in Ragwe village [30].

### Selection of houses and intervention with bed nets

Preliminary mosquito collection was done at the residential houses in Ragwe village and 15 houses, each with a relatively high mosquito density were selected. The houses had standard structures and sizes with eaves or gaps between the top of the wall and the roof, which was common in African houses. The number of people sleeping in bedrooms and living rooms, the number of bed nets (Olyset Net and Permanet) used before intervention, and the number of Olyset Net, Olyset Duo, and PPF-impregnated net after intervention are shown in Table 1.

**Table 1:** Information on the houses in which bed net interventions were conducted.

House No.	No. of people sleepinga)		No. of bed nets used before intervention		No. of bed nets used after intervention		
	Bedroom	Living room	Olyset Net	Permanet	Olyset Net (Oly)	Olyset Duo (Duo)	Pyriproxyfen (PPF)
RAG002	2A	3C	0	3	2	-	-
RAG004	2A	1A+1C	2	0	-	3	-
RAG005	2A	0	2	0	-	-	2
RAG007	2A	3C	2	0	2	-	-
RAG008	2A	2C	2	0	-	2	-
RAG009	3A	3C	4	0	-	-	3
RAG010	1A	1C	0	2	2	-	-
RAG011	3A	4C	2	0	-	4	-
RAG014	1A	2C	1	0	-	-	2
RAG020	1A	1A+1C	0	2	1	-	-
RAG023	1A	1C	1	0	-	2	-
RAG027	2A	1C	2	0	-	-	2
RAG028	2A	4C	2	0	2	-	-
RAG029	1A	6C	1	0	-	1	-
RAG030	2A	0	1	1	-	-	2

a) A, adults (age>15 years); C, children (age ≤15 years).

New bed nets were supplied and hung in houses in sufficient numbers to cover all residents while sleeping. The distance between each house was 100–500 m. For each type bed net intervention, 5 houses were used. The house residents were informed about the study and their written consent was obtained before conducting the interventions on April 27 and 28, 2013.

### Mosquito collection

Pre- and post-intervention mosquito collections were done in the morning (07∶00–09∶00) by three groups consisting of three staffs each, for ca. 20 min per house. Three clusters consisting of five houses each in the vicinity were arranged and three collection groups rotated the clusters daily. Mosquito collections were performed four times prior to intervention (April 22, 24, 25, and 26, 2013) and four times after intervention (April 29, 30, May 1, and 2, 2013). Anopheline mosquitoes resting on the walls, under furniture, or anywhere else in the house were collected using a battery-powered aspirator (C-cell aspirator, BioQuip Products, CA, USA). The nozzles and containers of the aspirators were changed for the collection in the houses with Olyset Duo and PPF-impregnated net to avoid contamination. The collected mosquitoes were examined microscopically in a laboratory to distinguish the *Anopheles gambiae* complex from other anopheline species, based on the identification keys of Gillies and Coetzee [31]. Individual species within *An. gambiae* s.l. were identified using the multiplex polymerase chain reaction (PCR) method described by Scott et al. [32].

### Observation of egg production, hatchability, and longevity of blood-fed females

After the collection of mosquitoes, freshly blood-fed female mosquitoes were individually held in a 20-mL glass vial with a small amount of water (1–2 mL) and damp filter paper as an oviposition substrate until egg production or death occurred. The presence and the number of eggs oviposited were recorded daily and the whole egg batch was transferred to a paper cup (ca. 50 mL) with water to allow hatching. A daily record of egg batch hatching was conducted. It required 7–21 days to complete the observation, since egg hatching did not take place simultaneously. After collection of eggs and bodies, mosquitoes were identified by PCR as previously described. Ohashi et al. reported that the mean longevity of *An. gambiae* s.s. females exposed to the 0.1% PPF-treated nets were 5.6 days [27]. The mean longevity of the blood-fed females contacted 1% PPF-treated nets was thought to be less than 5.6 days. On the other hand, most blood-fed mosquitoes normally lay eggs in less than 4 days after blood meals and they sometimes die after oviposition. Therefore, in the present trial, the deaths of field-collected blood-fed females within 3 days were judged as the deaths caused by the effect of PPF and the comparison of the mortality between PPF-exposed mosquitoes (Olyset Duo and PPF-impregnated net) and unexposed ones (Olyset Net) was done.

### Detection of Point Mutations in the Voltage-Gated Sodium Channel in *An. gambiae* s.s

PCR and direct DNA sequencing were performed to identify point mutations at 1014L in the field-collected mosquitoes according to the method by Kawada et al. [10], [11]. Direct DNA sequencing was performed using the 3730 DNA Analyzer (Applied Biosystems). The electropherogram of the targeted amino acid replacement was analyzed using MEGA 4.0 public domain software. The unique DNA haplotype sequences were deposited into the GenBank.

### Data analysis

A generalized linear mixed model (GLMM) using the Poisson distribution was used to examine the number of mosquitoes collected before and after bed net intervention by using the package lme4 in R (http://www.R-project.org). The dates of collection and the houses were used as random effects. Comparisons of the total number of blood-fed and unfed females, the total number of dead and alive blood-fed females within 3 days after collection, and the total number of oviposited and non-oviposited blood-fed females before and after intervention of bed nets were performed with χ^2^ test using JMP 7.0J (SAS Institute Japan Inc., Tokyo, Japan). Kruskal–Wallis one-way analysis of variance was used to compare the number of oviposited eggs, hatchability, and the number of progenies before and after intervention.

### Ethics Statement

The protocol for the study (Case No. 2522) was approved by the Scientific Steering Committee and the National Ethics Review Committee of the Kenya Medical Research Institute. All necessary permits were obtained for the described field studies. No mosquito collection was done without the approval of the head of the village and the owner and occupants of the collection house.

## Results

### Effect of Olyset Net, Olyset Duo, and PPF-impregnated net on the number of collected female *An. gambiae* s.s

**Figure 1:**
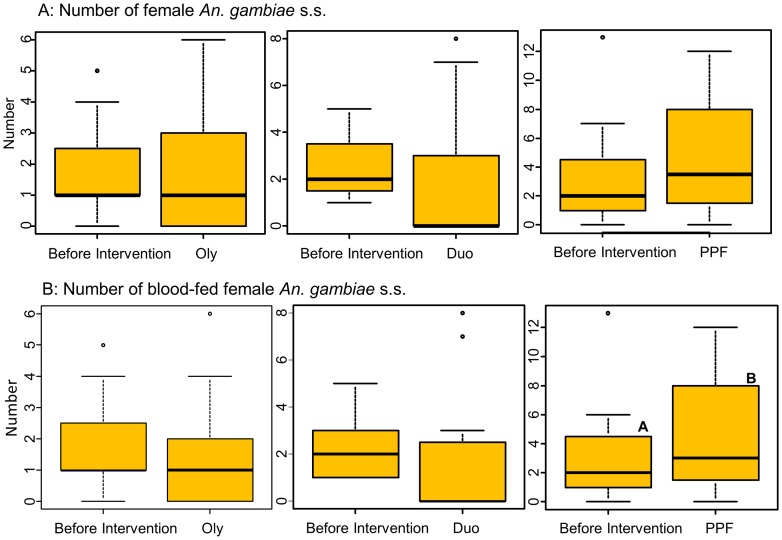
Box-and-whisker plots (medians: thick lines, with inter-quartile ranges) of the number of *Anopheles gambiae* s.s. females collected in the houses with Olyset Net (Oly), Olyset Duo (Duo), and PPF-impregnated net (PPF), before and after intervention. A, Average number of female *An. gambiae* s.s. females collected/house/day; B, Average number of blood-fed *An. gambiae* s.s. females collected/house/day. Different letters indicate significant difference by GLMM analysis.

One hundred and forty-five female *An. gambiae* s.s. and 5 female *An. arabiensis* were collected before the intervention, while 153 *An. gambiae* s.s. and 6 *An. arabiensis* were collected afterward. The allelic frequency of the point mutation in the voltage-gated sodium channel (L1014S) in *An. gambiae* s.s. collected was 98.4% (n = 96, Accession AB776705, AB776706). Average numbers of *An. gambiae* s.s. females collected in the houses with Olyset Net, Olyset Duo, and PPF-impregnated net were 1.90, 2.37, and 3.26 per house/day before intervention, and 1.56, 1.55, and 4.70 per house/day after intervention, respectively. Generalized linear mixed model for the number of collection revealed that net-by-intervention (before and after) interaction (P = 0.0047) was significant, while net (P = 0.083) and intervention (P = 0.740) were not significant. Significant difference was not observed in the number of mosquitoes collected in the houses with Olyset Net, Olyset Duo, and PPF-impregnated net (P>0.1 for Olyset Net and Olyset Duo, and P = 0.092 for PPF-impregnated net; Figure 1). Average numbers of blood-fed *An. gambiae* s.s. females collected in the houses with Olyset Net, Olyset Duo, and PPF-impregnated net were 1.85, 2.26, and 3.16 per house/day before intervention, and 1.33, 1.45, and 4.35 per house/day after intervention, respectively. Generalized linear mixed model for the number of collection revealed that both net (P<0.001) and net-by-intervention (before and after) interaction (P = 0.0089) were significant, while intervention (P = 0.891) was not significant. Decreased number of blood-fed females were observed in the houses with Olyset Net and Olyset Duo, although these differences were not significant (P>0.1), while a significant increase was observed in the houses with PPF-impregnated net (P<0.0001; Figure 1).

### Effect of Olyset Net, Olyset Duo, and PPF-impregnated net on blood feeding, longevity of blood-fed females, and oviposition of *An. gambiae* s.s

**Figure 2:**
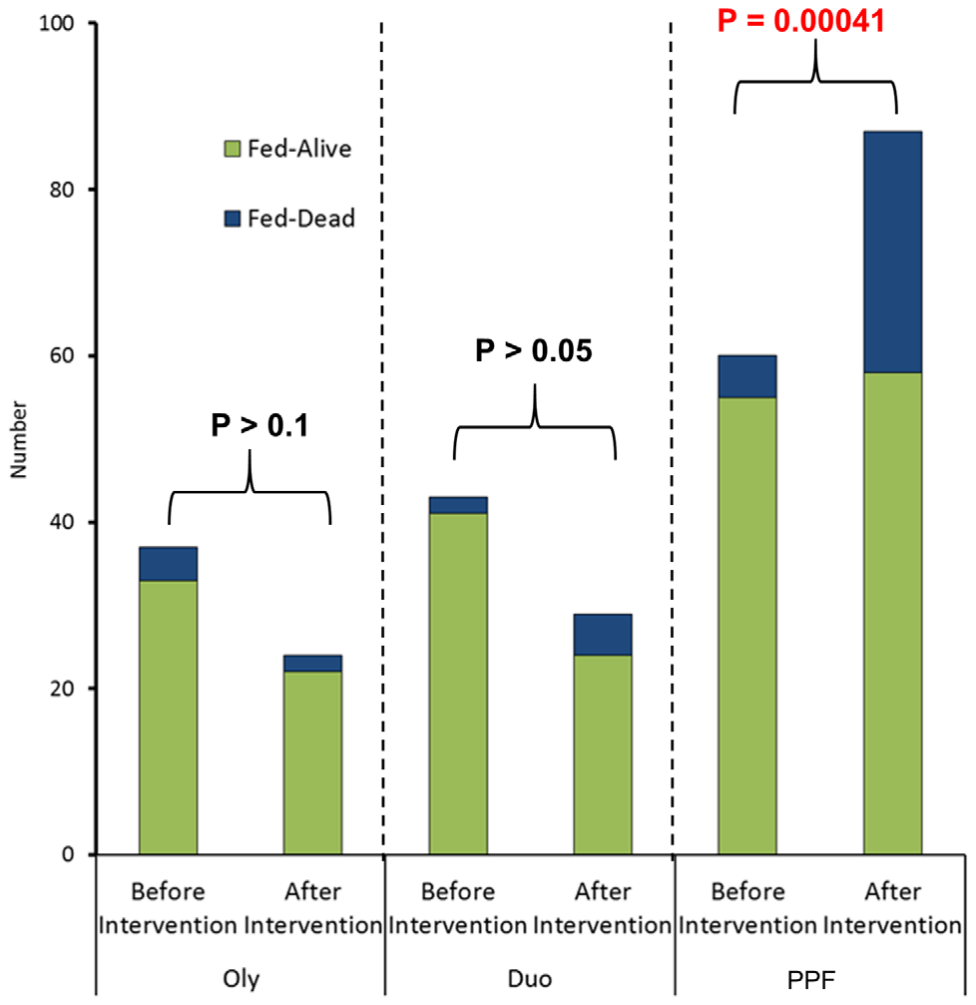
Total number of dead (within 3 days after collection) (Fed-Dead) and alive (Fed-Alive) blood-fed females of *Anopheles gambiae* s.s. before and after intervention with Olyset Net (Oly), Olyset Duo (Duo), and PPF-impregnated net (PPF). Figures indicate the P-value by χ^2^ test.

Mortality (3 days after collection) of the females in the houses with Olyset Net, Olyset Duo, and PPF-impregnated net were 10.8%, 4.7%, and 8.3% before intervention, and 8.3%, 17.2%, and 33.3% after, respectively. The difference in the proportion of the total number of dead blood-fed females against the total number of live blood-fed females of *An. gambiae* s.s. before and after intervention of PPF-impregnated net was significant (P = 0.00041), while those in the houses with Olyset Net (P>0.1) and with Olyset Duo (P>0.05) were not significant (Figure 2).

**Figure 3:**
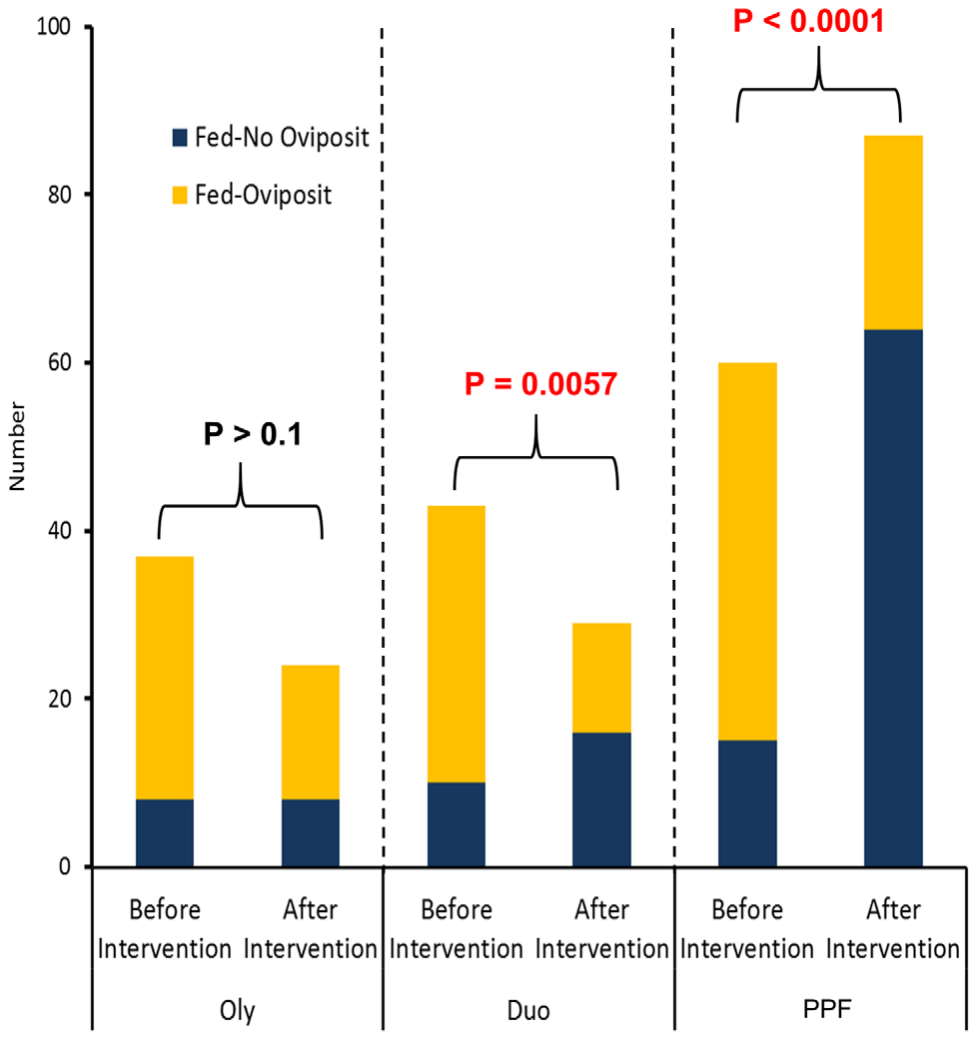
Total number of oviposited (Fed-Oviposit) and non-oviposited (Fed-No Oviposit) blood-fed females of *Anopheles gambiae* s.s. before and after intervention with Olyset Net (Oly), Olyset Duo (Duo), and PPF-impregnated net (PPF). Figures indicate the P-value by χ^2^ test.

Percentage of the blood-fed females ovipositing in the houses with Olyset Net, Olyset Duo, and PPF-impregnated net were 78.4%, 76.7%, and 75.0% before intervention, and 66.7%, 44.8%, and 26.4% after, respectively. Significant differences in the proportion of the total number of ovipositing blood-fed females vs. the total number of non-ovipositing blood-fed females of *An. gambiae* s.s. before and after intervention were observed in the houses with Olyset Duo (P = 0.0057) and PPF-impregnated net (P<0.0001), while that in the houses with Olyset Net was not significant (P>0.1) (Figure 3).

### Effect of Olyset Net, Olyset Duo, and PPF-impregnated net on the fecundity of *An. gambiae* s.s

**Figure 4:**
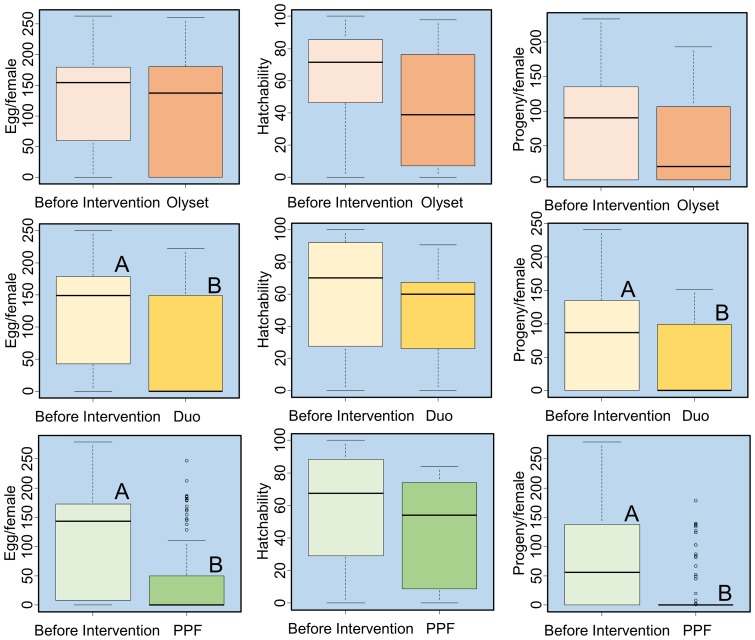
Box-and-whisker plots (medians: thick lines, with inter-quartile ranges) of the number of eggs oviposited, hatchability of eggs, and number of progenies produced by *Anopheles gambiae* s.s. females collected from houses with Olyset Net (Oly), Olyset Duo (Duo), and PPF-impregnated net (PPF), before and after intervention. Different letters indicate significant difference by Kruskal-Wallis one-way analysis of variance.

Average numbers of eggs oviposited before intervention in the houses with Olyset Net, Olyset Duo, and PPF-impregnated net were 124.6, 126.9, and 118.1/female and those after intervention were 111.8, 66.1, and 39.5/female, respectively. The difference in the number of oviposited eggs among the pre-intervention and post-intervention houses was significant (P = 0.0008), and higher and significant reduction in the number of eggs were observed in the houses where Olyset Duo (P = 0.0083) and PPF-impregnated nets (P<0.0001) were used compared to that in the pre-intervention houses (Figure 4).

Average hatchability of eggs oviposited by the blood-fed *An. gambiae* s.s. females collected before intervention in the houses with Olyset Net, Olyset Duo, and PPF-impregnated net was 60.9%, 59.3%, and 57.9% and those in the houses with Olyset Net, Olyset Duo, and PPF-impregnated net after intervention were 42.6%, 51.3%, and 42.9%, respectively. Hatchability in the pre-intervention houses and post-intervention houses was not significant (P = 0.852) (Figure 4).

Average numbers of progeny produced by the blood-fed *An. gambiae* s.s. females collected before intervention in the houses with Olyset Net, Olyset Duo, and PPF-impregnated net was 79.9, 77.2, and 72.6/female and those in the houses with Olyset Net, Olyset Duo, and PPF-impregnated net after intervention were 54.7, 38.4, and 19.6/female, respectively. Difference in the number of progenies between the pre-intervention houses and post-intervention houses was significant (P = 0.0021), and considerably higher reduction was observed in the houses in which Olyset Duo (P = 0.0195) and PPF-impregnated nets (P<0.0001) were used compared to that in the pre-intervention houses (Figure 4).

## Discussion

The *An. gambiae* s.s. population in the study area was pyrethroid-resistant governed by a point mutation of the voltage-gated sodium channel (L1014S) at high frequency, as previously reported [9], [33]. Additionally, a reduced repellency to pyrethroids has been reported in *An. gambiae* s.s. population in the study area as compared to the other malaria vectors, *An. arabiensis* and *An. funestus* s.s. [33]. This population attacked humans mainly at midnight irrespective of the use of LNs [34]. Therefore, we hypothesized that almost all blood-fed females had contact with the bed nets before or after the blood feeding, although the frequency of contact or total duration of contact was not measured in the present study.

An increase in the number of collected mosquitoes were observed in the houses in which PPF-impregnated nets were used. Further, some blood-fed mosquitoes were observed resting inside, as well as outside the PPF-impregnated bed nets, while no mosquitoes were collected from inside the permethrin-incorporated Olyset Net and Olyset Duo. This indicated that permethrin played a role in reducing the mosquito numbers inside the houses. No significant difference was noted in the number of blood-fed and unfed females of *An. gambiae* s.s. before and after intervention in the houses with Olyset Net, Olyset Duo, and PPF-impregnated nets were used. Since the present study was performed in houses where the residents had no restrictions on the sleeping times, some might have been outside the bed nets during midnight or might have slept under the bed nets in a manner that could have resulted in receiving mosquito bites.

Mortality (within 3 days after collection) of the blood-fed females in the houses with Olyset Duo and PPF-impregnated net increased from 4.7% to 17.2% and from 8.3% to 33.3%, respectively, after intervention. Significant differences were noted in the proportion of the number of dead blood-fed females against the number of live blood-fed females before and after intervention in the houses with PPF-impregnated nets. This increase in mortality clearly indicated the effect PPF had on the longevity of blood-fed females as previously reported [27]. Similarly, methoprene was reported to cause mortality to *Ae. aegypti* at 24 h following blood consumption [22]. Damages to internal tissues such as Malpighian tubules, midguts, and salivary gland cells have been observed in PPF-treated cat flea *Ctenocephalides felis* (Bouché), as reported by Meola et al. [35], and similar damages might have caused the deaths in the mosquitoes. Additionally, the percentage of the blood-fed females oviposited in the houses with Olyset Duo and PPF-impregnated nets reduced from 76.7% to 44.8% and from 75.0% to 26.4%, respectively, after intervention. Significant differences in the proportion of ovipositing, blood-fed females before and after intervention were observed in the houses with Olyset Duo and PPF-impregnated nets. The mortality rate in 3 days and the oviposition of the blood-fed females might be one of the means to determine the efficacy of PPF-impregnated bed nets in a field study, since the observation of egg production and hatchability requires time, special apparatus such as binoculars, is costly, and requires technical skill.

The number of oviposited eggs and progenies were significantly reduced in Olyset Duo and PPF-impregnated nets as compared to those before intervention. There was no difference in hatchability. In the current study, the same materials were used for Olyset Net and bed nets in which 1% PPF was impregnated. In the laboratory study using the laboratory-bred colony of *An. gambiae* s.s. (insecticide-susceptible ICIPE strain having 6.3% allelic L1014S mutation [9]), more than 90% reduction in offspring/blood-fed female was observed by the 3-min exposure to the same net materials (unpublished data). Ohashi et al. reported 100% reduction in fecundity of *An. gambiae* s.s. upon 3-min contact to the nets dipped in 0.01% isopropyl alcohol solution of PPF [27]. Kawada et al. reported that the minimum dosage of PPF, which causes sterility in female houseflies, was 5 µg per female [25]. On the other hand, 0.05–0.49 µg per female of PPF was thought to be effective in *Ae. aegypti*, since these amounts were detected in the mosquitoes that had reduced fecundity [24]. Even though the amount of PPF picked up by the mosquito after a short contact time with the PPF-impregnated net surface was not known, we believe it could be within the above range.

PPF has been reported to be effective against DDT, dieldrin, organophosphate, and carbamate-resistant anopheline mosquitoes as a larvicide, indicating the absence of cross-resistance of PPF to these insecticides [36]. However, only few studies have shown the relationship between pyrethroid-resistance and efficacy of PPF in mosquitoes. Kasai et al. suggested cross-resistance between etofenprox and insect growth regulators (IGRs) such as diflubenzuron and PPF in *Culex pipiens pipiens*, which were found in Japan [37]. There does not seem to be no other reports studying the above issue. Absence in cross resistance of PPF to other insecticides, especially pyrethroids, is essential for the selection of PPF as an active ingredient for LNs because of the aforementioned serious resistance problems in malaria vectors. The present study showing that the high sterilizing efficacy of PPF against pyrethroid-resistant *An. gambiae* s.s. might provide a solution to the above issue.

The current study also demonstrated the impact of permethrin and PPF when used in combination. The use of only PPF in a bed net might increase the risk of mosquito bites even though the mosquitoes that came in contact with the net might later have died (due to the impact of PPF on longevity) or have been sterilized. However, permethrin has an excito-repellent activity to mosquitoes that might reduce the contact chance of mosquitoes to bed net surface. Siegert et al. reported in a recent study that Olyset Net reduced the landing attempts of mosquitoes and elevated their flight frequency, resulting in low incidence of mortality [38]. Combined use of permethrin and PPF, therefore, might be antagonistic. The current study, however, demonstrated the effectiveness of permethrin incorporated PPF bed net (Olyset Duo) in reducing the number of ovipositing females and the number of eggs laid, and suppressing progeny through these mechanisms. The fact that a combination of a repellent pyrethroid and PPF was effective in sterilizing mosquitoes might be partly due to the reduced repellency of permethrin to *An. gambiae* s.s. in the study area [33]. Further, high anthropophily and midnight feeding habits [34] might have contributed positively to the effect of PPF. Recently, Ngufor et al. conducted the experimental hut trial of Olyset Duo against pyrethroid resistant laboratory colonies of *An. gambiae* s.s. [39]. The authors concluded that the pyrethroid-resistant mosquitoes which fail to be killed by permethrin could be sterilized if the net also contains PPF, resulting in greater reductions in the abundance of pyrethroid resistant malaria vectors.

The observation of oviposition and hatchability of mosquitoes requires skills, apparatus (binoculars and other equipment), and long term incubation of eggs, since it takes 1–3 weeks for the completion of egg hatching because egg hatching does not take place simultaneously [40]. We could not use large number of houses in the present study because of the limited manpower and apparatus. Larger scale field studies with sufficient number of skilled staffs and apparatus will be required for the further comprehension of the effectiveness of PPF. Further studies on the other wild pyrethroid-resistant mosquito populations such as *An. arabiensis* and *An. funestus* s.s. will provide a more convincing explanation to the above suggestions. Selection of PPF concentration, which would give the maximum efficacy while being cost-effective, as well as selection of the optimum concentration of permethrin or selection of another insecticide that can substitute for permethrin are issues of interest for future studies.
